# Mechanisms and therapeutic prospects of short-chain fatty acids in neonatal hypoxic–ischemic encephalopathy via modulation of mitochondrial dysfunction and endoplasmic reticulum stress

**DOI:** 10.3389/fnmol.2026.1875196

**Published:** 2026-10-12

**Authors:** Yinyu Wang, Lingjuan Gao, Yu Xu, Limei Niu, Xuan Tang, Yunxu Zhou, Xianhui Deng, Zhidan Bao

**Affiliations:** 1 Department of Neonatology, Jiangyin People’s Hospital, Jiangyin, Jiangsu, China; 2 Department of Neurosurgery, Jiangyin Clinical College of Xuzhou Medical University, Jiangyin, Jiangsu, China; 3 School of Medicine and Health Sciences, Wuxi Taihu University, Wuxi, Jiangsu, China; 4 Wuxi Key Laboratory of Biomaterials for Clinical Application, Jiangyin, Jiangsu, China

**Keywords:** endoplasmic reticulum stress, gut–brain axis, mitochondrial dysfunction, neonatal hypoxic–ischemic encephalopathy, neuroprotection, short-chain fatty acids

## Abstract

Neonatal hypoxic–ischemic encephalopathy (HIE) is a common severe neurological disorder following perinatal asphyxia and a leading cause of neonatal mortality and long-term neurodevelopmental impairments, including cerebral palsy, epilepsy, and cognitive and behavioral deficits. It remains one of the primary causes of death and disability in newborns, imposing a substantial burden on families and society. Although therapeutic hypothermia is currently recognized as an effective intervention, its narrow treatment window, limited patient eligibility, and the persistence of significant sequelae in some affected infants underscore the urgent need for adjunctive therapeutic strategies targeting novel mechanisms. In recent years, short-chain fatty acids (SCFAs), which are metabolites of the gut microbiota, have garnered considerable attention due to their neuroprotective effects via the gut–brain axis. This article systematically reviews the potential mechanisms and therapeutic prospects of SCFAs in neonatal HIE, focusing on their modulation of mitochondrial dysfunction and endoplasmic reticulum stress (ERS).

## 1 Introduction

Neonatal hypoxic–ischemic encephalopathy (HIE) is a leading cause of death and long-term neurodevelopmental deficits, including cognitive impairment, cerebral palsy, epilepsy, and neurosensory disorders (Arnautovic et al., 2024; Dumbuya et al., 2021). Although therapeutic hypothermia (TH) is the current standard of care, its benefits have been predominantly observed in mild HIE, whereas predicting outcomes in moderate-to-severe cases remains challenging (Wang et al., 2023; Rao et al., 2022; Rodrigues et al., 2025). The secondary injury cascade following hypoxic–ischemic insult involves a deleterious interplay among excitotoxicity, oxidative stress, neuroinflammation, and apoptosis, wherein ATP depletion causes excessive glutamate release and NMDA receptor overactivation, leading to pathological Ca^2+^ influx, which subsequently induces mitochondrial reactive oxygen species (ROS) generation and activates calcium-dependent enzymes, eventually resulting in oxidative stress (Ginet et al., 2014; Qin et al., 2019; Kong and Lu, 2024). ROS further propagate neuroinflammation by driving microglial activation and the release of pro-inflammatory cytokines (TNF-α, IL-1β) via NF-κB, which in turn amplify glutamate and ROS release in a positive feedback loop (Zheng et al., 2022; Fang et al., 2024). Ultimately, both the mitochondrial (Bax/cytochrome c/caspase-9) and death receptor (TNF-α/caspase-8) pathways converge on caspase-3, triggering apoptosis and irreversible loss of neurons and oligodendrocyte precursor cells (Lu et al., 2024b; Feng et al., 2024; Wang et al., 2021). Mounting evidence highlights the role of the gut–brain axis in HIE, whereby gut microbiota dysbiosis exacerbates long-term cognitive impairment by promoting gut dysfunction and neuroinflammation. Short-chain fatty acids (SCFAs), chiefly acetate, propionate, and butyrate—gut microbiota-derived metabolites of dietary fibre—can influence the central nervous system through either the circulatory route or the vagus nerve, and have been demonstrated to balance mitochondrial dynamics and mitigate mitochondrial dysfunction, thus attenuating oxidative stress (Dong and Cui, 2022; You et al., 2025; Kassan et al., 2023). In neurological disorders, acetate activates pyruvate carboxylase to restore glycolysis and the TCA cycle; propionate preserves mitochondrial homeostasis in Alzheimer’s disease models; and butyrate enhances cerebral metabolism under both normoglycemic and hyperglycemic conditions, thereby conferring neuroprotection against stroke (He et al., 2024; Wang Z. et al., 2025; Holstein et al., 2025). In neonatal HIE, SCFAs improve motor function and attenuate microglial activation and neuroinflammation, though the precise mechanisms remain incompletely understood (Huang et al., 2026; Zhao et al., 2022; Ziabska et al., 2025). Given their low molecular weight, blood–brain barrier permeability, and favourable safety characteristics, SCFAs constitute a promising novel therapeutic avenue for neonatal HIE. Moreover, strategies aimed at modulating the gut microbiome to boost endogenous SCFA levels—for instance, through butyrate-producing bacteria or prebiotics—offer an equally attractive approach. In this review, we concentrate on the multi-target neuroprotective actions of SCFAs in neonatal HIE, with particular emphasis on their synergistic mitigation of mitochondrial dysfunction and endoplasmic reticulum stress.

## 2 Mitochondrial dysfunction and endoplasmic reticulum stress in hypoxic–ischemic encephalopathy: two interrelated initiating factors of neural injury

HIE, a severe form of brain injury resulting from perinatal asphyxia, is pathophysiologically centered on disrupted energy metabolism and overactivation of programmed cell death cascades (Wang X. et al., 2025; Hu et al., 2025; Zhao et al., 2025). Unless otherwise indicated, the mitochondrial dysfunction and endoplasmic reticulum (ER) stress discussed in Sections 2–4 refer to changes in the injured brain, particularly in neurons and glial cells, with additional effects in the neurovascular unit. Intestinal mitochondrial dysfunction and ER stress may also occur after systemic hypoperfusion, but are considered here as upstream contributors to gut barrier impairment and altered SCFA availability rather than as the same local pathological process. Within the brain, mitochondrial and ER dysfunction are concurrently triggered by hypoxic–ischemic (HI) insults and form a synergistically amplifying injury network.

### 2.1 Common upstream triggering mechanisms

Hypoxic–ischemic (HI) insult leads to oxygen–glucose deprivation (OGD), which first induces rapid ATP depletion and consequently causes failure of ATP-dependent ion pumps (Na^+^/K^+^-ATPase, SERCA, and PMCA), resulting in concurrent cytosolic calcium overload and ER calcium depletion (Liiv et al., 2024). Meanwhile, due to insufficient oxygen, electron leakage from the electron transport chain elicits a burst of ROS production; in parallel, calcium overload suppresses the activities of antioxidant enzymes, further aggravating oxidative stress. These three concurrent insults—ATP depletion, calcium homeostasis disruption, and oxidative stress—simultaneously impact both mitochondria and the ER, serving as a common “igniter” that primes dysfunction in both organelles.

### 2.2 Downstream effects of mitochondria

As a critical organelle possessing the dual identity of both an “energy powerhouse” and a “death switch” (Kong and Lu, 2024; Li et al., 2025), mitochondria, under normal conditions, perform four major physiological functions: ATP synthesis (providing >90% of neuronal energy), calcium homeostasis regulation, ROS production and scavenging, and apoptosis regulation (Cheng et al., 2022; Dong et al., 2023; Verma et al., 2022). However, under HI conditions, mitochondria rapidly shift from “cellular guardians” to “damage amplifiers.”

#### 2.2.1 Energy crisis

Compromised oxidative phosphorylation precipitates a drastic reduction in ATP production; dysfunction of the Na^+^/K^+^-ATPase triggers depolarization and extensive glutamate efflux, while the inadequate compensatory effect of anaerobic glycolysis, together with lactate accumulation-induced acidosis, directly inflicts injury upon mitochondrial membrane complexes (Sun et al., 2025; Scacco et al., 2025).

#### 2.2.2 Calcium overload

The dissipation of mitochondrial membrane potential not only impairs mitochondrial sequestration of cytosolic calcium but also, via the lowered membrane potential, induces mPTP opening, leading to additional calcium influx into the matrix and perpetuating a vicious cycle; this in turn activates hydrolases, resulting in disruption of cristae architecture (Ren et al., 2025; Angelova et al., 2025; Donnelly et al., 2024; Endlicher et al., 2023).

#### 2.2.3 Oxidative stress

Substantial ROS production arising from electron leakage, coupled with MnSOD depletion-induced impairment of ROS scavenging, results in oxidative assault on membrane lipids, DNA, and respiratory chain proteins; this insult is further exacerbated by a secondary ROS burst during the reperfusion phase (Okoye et al., 2023; Tan et al., 2023; Botting-Lawford et al., 2025; Yang X. et al., 2025).

#### 2.2.4 Apoptosis initiation

Opening of the mPTP, together with Bax/Bak conformational rearrangements, triggers mitochondrial outer membrane permeabilization (MOMP), resulting in the release of cytochrome c and apoptosis-inducing factor (AIF), among other pro-apoptotic factors; these events subsequently engage both the caspase cascade and caspase-independent DNA degradation machinery, culminating in the onset of irreversible apoptosis (Zhao et al., 2023; Jiang et al., 2018; Rodriguez et al., 2018; Zhu et al., 2007).

### 2.3 Downstream effects of the endoplasmic reticulum

Under hypoxic–ischemic (HI) conditions, the ER—a key organelle governing protein synthesis, folding, and calcium sequestration—is likewise exposed to the aforementioned triple insults (Fehr et al., 2025; Mao et al., 2025). ATP depletion impairs the function of ER luminal chaperones such as GRP78, calcium depletion compromises the protein-folding milieu, and ROS not only oxidize ER membrane lipids but also suppress SERCA activity; these converging insults jointly precipitate ER stress (Wiseman et al., 2022; Yang R. et al., 2025; Singh et al., 2025; He et al., 2023). In response to these insults, neurons activate the unfolded protein response (UPR), a signaling cascade that displays a dichotomous “double-edged sword” nature—capable of both adaptive protection and pro-apoptotic signaling.

#### 2.3.1 Adaptive UPR (pro-survival)

Activation of the protein kinase RNA-like ER kinase (PERK)-eukaryotic initiation factor 2 alpha (eIF2α) axis suppresses general protein synthesis, while activating transcription factor 4 (ATF4) supports adaptive antioxidant responses, including nuclear factor erythroid 2-related factor 2 (NRF2)-dependent expression of heme oxygenase-1, NAD(P)H quinone dehydrogenase 1, and glutathione-synthesis genes. Inositol-requiring enzyme 1 alpha (IRE1α)-mediated X-box binding protein 1 (XBP1) splicing enhances molecular chaperone expression and ER-associated degradation (ERAD); activating transcription factor 6 (ATF6) converges on these adaptive targets. These integrated signals can reduce the folding burden and promote ER recovery (Mao et al., 2025; Cai et al., 2025; Carloni et al., 2014; Zhong et al., 2024).

#### 2.3.2 Apoptotic UPR (pro-death)

Under excessive or prolonged ER stress, hyperactivation of PERK increases C/EBP homologous protein (CHOP), which suppresses B-cell lymphoma 2 (Bcl-2) while inducing Bcl-2-interacting mediator of cell death (Bim) and p53 upregulated modulator of apoptosis (PUMA), further aggravating oxidative stress. Concurrently, sustained IRE1α activation engages apoptosis signal-regulating kinase 1 (ASK1), resulting in c-Jun N-terminal kinase (JNK)/p38 pathway activation. Following proteolytic cleavage and activation, caspase-12 cleaves caspase-9 and triggers a mitochondria-independent apoptotic cascade. Thus, elevated CHOP and activated caspase-12 can commit injured brain cells to apoptosis (Mao et al., 2025; Hu et al., 2024; Zhang et al., 2021; Wang et al., 2016; Zhang et al., 2017).

## 3 Vicious cycle: MAM-mediated interplay between mitochondria and the ER exacerbates injury

Far from operating independently, mitochondria and the ER maintain intimate communication via a specialized physical and functional platform designated as mitochondria-associated ER membranes (MAMs) (Jiang et al., 2023; Mao et al., 2022). MAMs represent specialized membrane contact sites at the ER–mitochondria interface, constituted by a repertoire of tethering proteins—including IP3R, Grp75, VDAC1, and PACS-2—that maintain stable intermembrane distances in the nanoscale range (≈10–30 nm). These junctions provide a pivotal platform that integrates lipid biosynthesis and trafficking, Ca^2+^ signaling within microdomains, and the targeted relay of ER stress signals toward mitochondria (Zhang et al., 2024; Xiao et al., 2025; Zhao and Sheng, 2025; Liu et al., 2025). Under physiological settings, MAMs orchestrate the spatiotemporally restricted transfer of Ca^2+^ from the endoplasmic reticulum to mitochondria, a process that activates rate-limiting enzymes of the tricarboxylic acid (TCA) cycle in the mitochondrial matrix and thereby maintains the efficacy of oxidative phosphorylation (Chen W. et al., 2024; Song et al., 2022; Xian et al., 2024). However, in the context of OGD and energy depletion resulting from HIE, this MAM-based interface serves as a core nexus that amplifies damaging signals and orchestrates a cascading vicious cycle as delineated below.

Hypoxia-ischemia results in the accumulation of unfolded/misfolded proteins within the ER lumen, while concurrent ATP depletion renders ATP-dependent molecular chaperones (e. g., Grp78/BiP and Grp94) and foldases (e. g., PDI) inactive within the ER lumen. In addition, energy deficiency compromises SERCA activity, thereby impairing ER calcium-pumping function. Acting in concert, these perturbations elicit pronounced ER stress (ERS) (Mao et al., 2025; Cai et al., 2025; Carloni et al., 2014).

In the state of ERS, IP3Rs are excessively open, and the inactivation of SERCA prevents the ER from re-sequestering calcium ions. Consequently, substantial Ca^2+^ is discharged from the ER into the cytosol, resulting in cytosolic calcium overload, while the ER itself becomes depleted of calcium (Wang et al., 2017). The excess cytosolic Ca^2+^ is directly channeled into the mitochondrial matrix through the upregulated IP3R-Grp75-VDAC1 complexes at the MAMs. This physical coupling converts the physiologically restricted calcium signal into sustained mitochondrial calcium overload (Mao et al., 2025; Carloni et al., 2014). Calcium overload in the mitochondrial matrix triggers the opening of the mitochondrial permeability transition pore (mPTP), which induces a sharp increase in inner membrane permeability, complete dissipation of the transmembrane potential, uncoupling of the electron transport chain (ETC), and cessation of oxidative phosphorylation, further diminishing ATP synthesis (Endlicher et al., 2023; Mendoza et al., 2024). mPTP opening and ETC dysfunction markedly increase electron leakage at respiratory chain complexes I and III; large numbers of electrons react with residual oxygen, triggering a burst production of superoxide anions, hydrogen peroxide, and hydroxyl radicals, thus forming an oxidative stress storm (Okoye et al., 2023; Botting-Lawford et al., 2025; Wang K. et al., 2025). These ROS not only attack the mitochondrial membrane lipids, DNA, and respiratory chain proteins per se, but also diffuse into the cytosol and ER. The excess mitochondria-derived ROS oxidize lipids and sulfhydryl groups on the ER membrane, interfere with disulfide bond formation, and directly suppress SERCA activity, preventing the ER from effectively re-sequestering calcium from the cytosol, which further exacerbates ER calcium depletion. Concurrently, ROS attack molecular chaperones in the ER lumen, causing their oxidative inactivation and continuously deteriorating the protein-folding environment (Tan et al., 2023; Yang X. et al., 2025; AVS et al., 2025). This cascade ensures that the initial ERS is not only unresolved but is also significantly amplified.

Persistent and aggravated ERS renders the UPR unable to sustain its compensatory function: hyperactivation of the PERK–eIF2α pathway leads to a marked upregulation of the pro-apoptotic transcription factor CHOP (GADD153). CHOP, on the one hand, suppresses the anti-apoptotic protein Bcl-2, and on the other hand, upregulates the pro-apoptotic proteins Bim and PUMA, while also depleting ER glutathione reserves (Mao et al., 2025; Hu et al., 2024). Concurrently, sustained IRE1α activation recruits ASK1 and activates the JNK/p38 MAPK pathways, further exacerbating mitochondrial outer membrane permeabilization (MOMP) (Zhang et al., 2021). In addition, caspase-12, located on the outer membrane of the ER, is cleaved and activated by calpain or the IRE1α–TRAF2 complex, which then specifically cleaves caspase-9, initiating a mitochondria-independent ER-specific apoptotic pathway (Wang et al., 2016; Zhang et al., 2017).

Collectively, these observations clearly indicate that MAMs function as bidirectional signaling relay hubs, coupling ERS and mitochondrial dysfunction into a positive feedback loop—ERS → calcium overload → mPTP opening → ROS burst → ER calcium depletion/chaperone inactivation → aggravated ERS—each step of which is either transmitted through or regulated by MAMs. During the acute phase of HIE, this vicious cycle continuously subjects neurons to the combined onslaught of energy failure, oxidative damage, and apoptotic signaling. Extensive studies have demonstrated that in HIE animal models, inhibiting mitochondrial ROS production or stabilizing MAM architecture significantly reduces CHOP and cleaved caspase-12 levels, thereby interrupting this vicious cycle. In the neonatal brain, apoptosis can persist for several days following hypoxic–ischemic injury, which represents a therapeutic opportunity during the delayed injury phase for intervention. By supplementing certain substances, such as SCFAs, to target MAMs or their upstream/downstream nodes, it holds promise for inhibiting neuronal apoptosis and attenuating hypoxic–ischemic injury.

## 4 Short-chain fatty acids as multi-target disruptors of the mitochondrial–ER vicious cycle

Short-chain fatty acids (SCFAs) are a class of saturated fatty acids consisting of 1 to 6 carbon atoms. In mammals, they are primarily derived from the anaerobic fermentation of dietary fiber by the gut microbiota in the colon (Kalyanaraman et al., 2024; Fusco et al., 2023). Among these, acetate, propionate, and butyrate are the most abundant, collectively accounting for over 90% of total SCFAs (Cong et al., 2022; Kondo et al., 2022). SCFAs are rapidly absorbed by epithelial cells in the colonic lumen; among them, butyrate serves as the primary energy substrate for colonic epithelial cells, with approximately 70% being utilized locally (Hamed et al., 2023; Peng et al., 2024), whereas acetate and propionate are predominantly transported via the portal vein into the systemic circulation, distributing to the liver, muscle, and central nervous system (CNS) (Song et al., 2023; Luo et al., 2025; Tao et al., 2023). SCFAs can partially cross the blood–brain barrier (BBB) and are present at low concentrations in the cerebrospinal fluid and brain parenchyma. Although their cerebral concentrations are far lower than those in the periphery, they are nevertheless sufficient to exert regulatory signaling functions (Chenghan et al., 2025; Rosell-Cardona et al., 2025; Tang et al., 2022). In recent years, owing to their multi-targeting properties and low toxicity, SCFAs have shown promising neuroprotective potential in models of HIE and other neurological injury models (Ziabska et al., 2025; Hermans et al., 2024; Gao et al., 2022; Si et al., 2025).

The core pathological mechanism of hypoxic–ischemic brain injury lies in the collapse of energy metabolism triggered by oxygen–glucose deprivation (OGD). The acute depletion of ATP not only leads to ion pump failure and disruption of calcium homeostasis, but also induces a burst of ROS production via ETC dysfunction, thus forming a “triple hit” comprising energy crisis, calcium overload, and oxidative stress. This triple hit simultaneously affects mitochondria and the ER, subjecting both organelles to dysfunction, which in turn propagates injury signals between each other through MAMs as a physical and functional interface, thereby generating a progressively amplifying vicious cycle. Within this framework, the actions of SCFAs should not be construed as independent parallel effects; rather, through three interrelated pathways—(a) epigenetic regulation (HDAC inhibition), (b) receptor-mediated signaling (GPR41/43), and (c) direct metabolic modulation—SCFAs exert targeted interventions at distinct nodes of the vicious cycle, synergistically dismantling this injury network.

### 4.1 Epigenetic regulation—HDAC inhibition: upregulation of PGC-1α and GRP78

One of the most well-recognized neuroprotective mechanisms of SCFAs is their function as endogenous histone deacetylase inhibitors (Pu et al., 2025; Meng et al., 2025; Inamoto et al., 2023). Among these, butyrate exhibits the most potent inhibitory activity, followed by propionate, whereas acetate is relatively weak. Consequently, the majority of current studies have centered on butyrate. Butyrate can traverse the nuclear envelope and competitively inhibit class I and class IIa histone deacetylases, thereby elevating the acetylation levels of histones H3/H4. This acetylation relaxes chromatin structure and facilitates the transcription of specific protective genes (Nshanian et al., 2025; Li et al., 2024).

In terms of intervention nodes within the vicious cycle, this pathway simultaneously targets both mitochondria and the ER at the transcriptional level, thereby augmenting the functional reserves of both organelles from the outset. First, via HDAC inhibition, SCFAs upregulate the expression of PGC-1α and its downstream target genes. As a master regulator of mitochondrial biogenesis, PGC-1α upregulation promotes the generation of nascent mitochondria, counteracting the decline in oxidative phosphorylation efficiency resulting from suppressed mitochondrial biogenesis following HI injury (Wu et al., 2024; Ye et al., 2024; Lu et al., 2024a). Second, butyrate epigenetically upregulates the expression of ER molecular chaperones, including GRP78 (BiP) and GRP94, as well as the foldase PDI, thereby enhancing ER protein-folding capacity (36244453). In HIE models, butyrate treatment also significantly upregulates the expression of brain-derived neurotrophic factor (BDNF) and glial cell line-derived neurotrophic factor (GDNF), while simultaneously enhancing the transcription of antioxidant enzyme genes such as superoxide dismutase (SOD2) and glutathione peroxidase (GPx) (Li et al., 2024; Zhao et al., 2025; Ziabska et al., 2026).

Collectively, these findings demonstrate that, through the single axis of HDAC inhibition, SCFAs concurrently address the two core pathological issues—mitochondrial “energy deficiency” and ER “folding overload”—thereby blocking the initiation of the vicious cycle at its source.

### 4.2 Receptor-mediated signaling via GPR41/43 activation: inhibition of NF-κB and the PERK–eIF2α–ATF4–CHOP Axis

SCFAs mediate rapid signal transduction through G protein-coupled receptors, primarily free fatty acid receptor 3 (FFAR3/GPR41) and free fatty acid receptor 2 (FFAR2/GPR43) (Rekha et al., 2024; Liu et al., 2024; Muralitharan et al., 2025). These receptors are expressed in brain-resident and neurovascular cells, although expression levels and downstream responses vary by cell type and experimental context (Park et al., 2019; Zhong et al., 2025; Lahariya et al., 2026).

In terms of intervention nodes within the vicious cycle, this pathway interrupts two key pro-injury signaling pathways—inflammatory signals and ER stress-mediated pro-apoptotic signals—at the signal transduction level, preventing their transmission to mitochondria.

On the one hand, upon binding to their receptors, SCFAs inhibit the nuclear translocation and transcriptional activity of the prototypical pro-inflammatory transcription factor NF-κB, reducing the release of TNF-α, IL-1β, IL-6, and other inflammatory cytokines, while promoting the expression of the anti-inflammatory cytokine IL-10 (Cao et al., 2025; Wang J. et al., 2025; Xiao et al., 2026). During the acute phase of HIE, the inflammatory storm triggered by excessive microglial activation is a major driver of secondary brain injury (Ding et al., 2026; Liu et al., 2022); SCFAs effectively curtail this response through the GPCR pathway (Burnsed et al., 2026; Xie et al., 2021). The attenuation of inflammatory signals indirectly reduces the oxidative burden on mitochondria and prevents further interference of ER function by inflammatory factors.

On the other hand—and more critically—SCFAs suppress the hyperactivation of the PERK–eIF2α–ATF4–CHOP pathway via GPR41/43, reducing the levels of the pro-apoptotic protein CHOP and cleaved caspase-12 (Chen Y. et al., 2024; Du et al., 2024; Qureshi et al., 2023). CHOP serves as a critical switch that shifts ER stress from an adaptive to an apoptotic response; its elevated expression suppresses Bcl-2, upregulates Bim/PUMA, exacerbates oxidative stress, and initiates mitochondrial outer membrane permeabilization (MOMP) and caspase-9-dependent apoptotic pathways. Intervention by SCFAs at this node essentially blocks the “death signal” transmitted from the ER to mitochondria, redirecting the UPR from an apoptotic to an adaptive trajectory.

### 4.3 Direct metabolic modulation: stabilizing membrane potential, inhibiting mPTP, and blocking IP3R-mediated ER calcium release

Following hypoxia-ischemia, mitochondrial biogenesis is suppressed, oxidative phosphorylation efficiency declines, membrane potential dissipates, the mPTP opens aberrantly, and cytochrome c is released, initiating apoptosis (Lu et al., 2024b; Feng et al., 2024). In terms of intervention nodes within the vicious cycle, this pathway directly targets three critical axes at the organelle interaction interface (MAM) and the metabolic level: First, SCFAs (notably butyrate) upregulate PGC-1α and its downstream target genes via HDAC activity, promoting the generation of nascent mitochondria (Wu et al., 2024; Ye et al., 2024; Lu et al., 2024a). Second, SCFAs regulate the NAD^+^/NADH ratio and SIRT1 activity, thereby enhancing respiratory chain complex activity, stabilizing membrane potential, and inhibiting mPTP opening (Yi et al., 2025; Gao et al., 2024), which directly prevents the release of cytochrome c and AIF, thereby blocking the initiation of the apoptotic cascade. Third—and potentially most critically—SCFAs block IP3R-mediated ER calcium release. A core function of MAMs is to directionally transmit calcium signals from the ER to mitochondria via the IP3R-Grp75-VDAC1 complex. Under HI conditions, ER calcium depletion and mitochondrial calcium overload form a positive feedback loop through this pathway. By stabilizing ER calcium stores and interfering with IP3R function, SCFAs attenuate calcium signaling transmitted via MAMs, thereby simultaneously alleviating both mitochondrial calcium overload and ER calcium depletion, breaking a key driving element of this vicious cycle.

The protective effect of SCFAs on BBB integrity is particularly critical in preventing vasogenic cerebral edema following HIE. SCFAs such as butyrate can act on brain microvascular endothelial cells to upregulate the expression of tight junction proteins (claudin-5, occludin, ZO-1) while inhibiting the activity of matrix metalloproteinases (MMP-2/9), thereby reducing basement membrane degradation (Chenghan et al., 2025; Wang et al., 2026; Naseri et al., 2026). In addition, the anti-inflammatory effects of SCFAs indirectly mitigate BBB damage mediated by pericytes and microglia (Riew et al., 2021; Chen et al., 2025; Wang et al., 2023). Animal studies have demonstrated that oral administration of SCFAs or supplementation with SCFA-producing probiotics significantly reduces Evans blue extravasation and alleviates cerebral edema in HIE mouse models (Gao et al., 2022; He et al., 2022; Fabres et al., 2025).

Although BBB protection does not directly act on the intracellular mitochondrial-ER vicious cycle, by maintaining BBB integrity, it reduces peripheral inflammatory cell infiltration and the entry of neurotoxic substances into the brain, thereby creating a stable internal environment for the central nervous system. This indirectly provides a “peripheral safeguard” for the above-mentioned intracellular intervention pathways, preventing the cycle from being refueled at the systemic level.

In summary, short-chain fatty acids, particularly butyrate, do not act via a single pathway but rather exert targeted interventions at multiple vulnerable nodes of the mitochondrial-ER vicious cycle: (a) through HDAC inhibition, they simultaneously upregulate PGC-1α (mitochondrial biogenesis) and GRP78 (ER folding capacity) at the transcriptional level, replenishing the functional reserves of both organelles at their source; (b) via GPR41/43 receptor activation, they suppress the NF-κB and PERK–eIF2α–ATF4–CHOP pathways, abrogating pro-apoptotic signals transmitted from inflammation and ER stress to mitochondria at the signal level; and (c) through direct metabolic modulation, they stabilize mitochondrial membrane potential, inhibit mPTP, and block IP3R-mediated ER calcium release, dismantling the pathological coupling between mitochondria and the ER at the MAM interface. These three pathways act synergistically at the source, signal, and interface levels, collectively disrupting the positive feedback mechanism of the vicious cycle.

Given that HIE is a complex pathological process involving excitotoxicity, oxidative stress, inflammatory responses, and apoptotic cascades, SCFAs—by virtue of their multi-targeting properties and low toxicity—simultaneously intervene in mitochondrial dysfunction, ER stress, and the vicious cycle linking them, demonstrating their potential as a novel class of neuroprotective agents that merits further translational research exploration.

### 4.4 Cell-specific actions of SCFAs in the injured brain

The proposed mitochondrial-ER mechanism is principally relevant to neurons, but the therapeutic response is not neuron-exclusive. In neurons, SCFAs may support mitochondrial bioenergetics, limit ROS accumulation, preserve ER calcium homeostasis, and reduce apoptotic signaling. In microglia, SCFAs can restrain inflammatory activation through receptor-mediated and epigenetic mechanisms, thereby reducing cytokine-driven secondary injury. In astrocytes, SCFAs may improve metabolic and antioxidant support and facilitate glutamate clearance, indirectly protecting neurons from excitotoxicity. Oligodendrocyte lineage cells are highly vulnerable to oxidative and mitochondrial injury in the immature brain; thus, preservation of redox balance and inflammatory control may protect white-matter development. Direct evidence for each cell type in neonatal HIE remains uneven, with the strongest experimental support currently for neuronal protection and microglial modulation. Accordingly, future studies should use cell-resolved approaches to distinguish direct SCFA effects from secondary consequences of reduced tissue injury (Dong and Cui, 2022; Ziabska et al., 2025; Ziabska et al., 2026; Li et al., 2025; Wei et al., 2023; Ortiz-Samur et al., 2025).

## 5 An integrated model: short-chain fatty acids reverse the positive feedback loop

From a pathophysiological perspective, the neuroprotective effects of SCFAs in neonatal hypoxic–ischemic encephalopathy (HIE) can be conceptualized as a tripartite integrated model of “upstream deficiency—midstream amplification—downstream reversal.” This model posits that HIE first induces gut microbiota dysbiosis, resulting in insufficient endogenous SCFA production and thus depriving the organism of basal neuroprotective support to the brain; subsequently, the mitochondrial–endoplasmic reticulum (mitochondria–ER, Mito-ER) axis, lacking SCFA-mediated regulation, establishes a continuously self-amplifying positive feedback loop that progressively exacerbates neuronal injury; ultimately, exogenous SCFAs, by simultaneously targeting the two critical nodes—mitochondria and the ER—restore energy metabolism and calcium homeostasis, thereby converting this pathological positive feedback into a protective negative feedback and achieving neuroprotection (see Figure 1).

**Figure 1:**
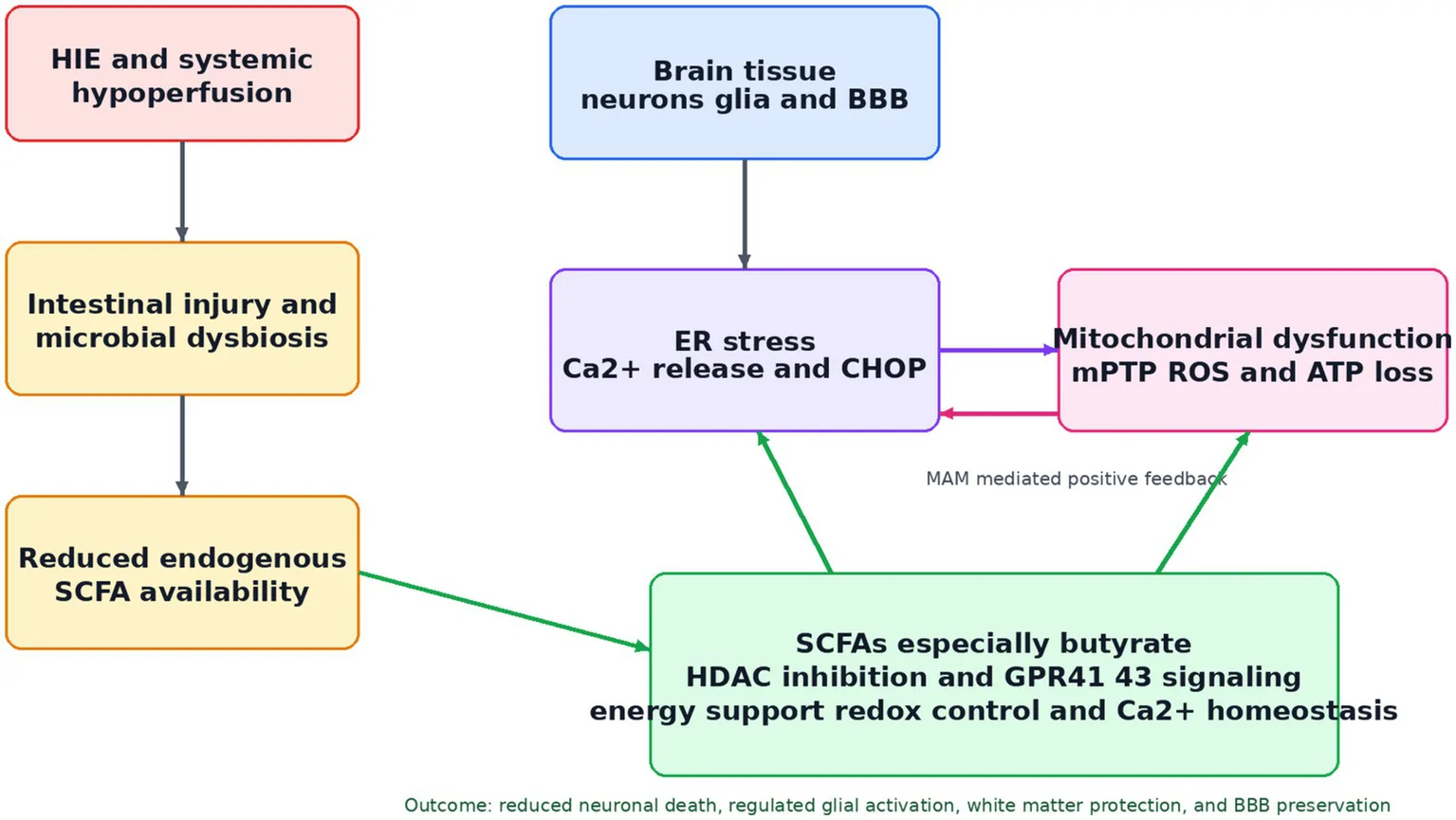
Proposed mechanism by which reduced endogenous SCFA availability and the brain Mito-ER positive-feedback loop contribute to neonatal HIE. SCFA-based interventions may act through epigenetic, receptor-mediated, and metabolic mechanisms to protect neurons, regulate glial responses, and preserve the blood–brain barrier. MAM, mitochondria-associated ER membrane; mPTP, mitochondrial permeability transition pore; ROS, reactive oxygen species.

### 5.1 Upstream: HIE injury induces gut microbiota Dysbiosis, reducing endogenous SCFA production and compromising basal neuroprotective reserves

Following the onset of HIE, systemic blood flow is prioritized to the brain and heart, resulting in reduced mesenteric perfusion, intestinal ischemia-hypoxia, mucosal barrier disruption, and microbial imbalance. The resulting loss of SCFA-producing obligate anaerobes may impair fermentation capacity and reduce endogenous SCFA availability (Huang et al., 2022; Gala et al., 2022). However, the immediate postnatal SCFA profile is dynamic: acetate is relatively dominant early after birth, whereas propionate and butyrate rise with feeding and microbial maturation (Duck et al., 2025; Lee et al., 2018). Therefore, the current evidence does not support a precise universal percentage decrease in SCFAs during the first 48 h of HIE. Future longitudinal studies should compare stool and plasma SCFAs with gestational age, feeding exposure, antibiotic use, therapeutic hypothermia, and injury severity taken into account.

This developmental timing also has therapeutic implications. Strategies that promote physiological SCFA production, such as human milk feeding, prebiotics, or carefully selected SCFA-producing probiotics, may be more suitable for longer-term restoration of the gut ecosystem than direct SCFA administration alone. However, their onset is not immediate and may be affected by intestinal perfusion, feeding tolerance, antibiotic exposure, and the baseline microbiota. Direct SCFA delivery and microbiota-directed approaches should therefore be regarded as complementary strategies whose safety, dose, route, and timing require dedicated neonatal studies.

This alteration not merely signifies a reduction in a class of metabolites, but more importantly represents the dismantling of the organism’s innate neuroprotective barrier. Under normal physiological conditions, SCFAs continuously maintain brain tissue homeostasis via the gut–brain axis, including inhibiting microglial hyperactivation, preserving blood–brain barrier integrity, promoting mitophagy, and improving cellular energy metabolism (Cho et al., 2024; Qiao et al., 2024; Sun et al., 2026). Thus, the endogenous SCFA deficiency resulting from HIE deprives the nervous system of its basal protective capacity against hypoxic–ischemic stress, effectively releasing a pre-existing “physiological brake” and creating conditions conducive to the sustained amplification of subsequent organellar damage.

### 5.2 Midstream: initiation of the mitochondrial–ER vicious cycle, formation of pathological positive feedback, and driving of neuronal apoptosis

Against the backdrop of deficient endogenous SCFA protection, the HIE-induced abnormality in mitochondrial–ER coupling rapidly transitions into a continuously self-amplifying positive feedback state, which constitutes the core mechanism driving the progressive amplification of brain injury.

Hypoxia-ischemia first induces rapid ATP depletion and disruption of ER Ca^2+^ homeostasis, with substantial and sustained accumulation of unfolded proteins that progressively converts the UPR from its initial adaptive protective role to a pro-apoptotic signaling cascade (Bensellam et al., 2016; Chen et al., 2018). Simultaneously, the ER continuously releases Ca^2+^ to mitochondria via MAMs, leading to mitochondrial Ca^2+^ overload, which subsequently triggers mPTP opening, a burst of ROS production, and cytochrome c release (She et al., 2025; Sosunov et al., 2022; Ramakrishna and Krishnamurthy, 2023).

More importantly, these mitochondrial insults in turn further exacerbate ER dysfunction. The reduction in ATP generation compromises the protein-folding capacity of ATP-dependent molecular chaperones such as GRP78, whereas the persistent accumulation of ROS further disrupts the ER redox microenvironment, promoting the continuous buildup of misfolded proteins and inducing exacerbated ER stress. This gives rise to a self-perpetuating pathological cycle: ER stress → aberrant Ca^2+^ release → mitochondrial injury → ATP depletion/ROS elevation → aggravated ER stress. This positive feedback loop maintains neurons in a state of sustained stress, ultimately activating the CHOP–caspase-12–caspase-9 apoptotic pathway and MOMP, leading to widespread neuronal apoptosis (Zhong et al., 2024; Guan et al., 2025; Ma et al., 2026). This vicious cycle is most active during the delayed brain injury phase occurring 6–72 h post-reperfusion, which is currently recognized as the most critical therapeutic window for intervention.

### 5.3 Downstream: exogenous SCFAs simultaneously act on mitochondria and the ER, reversing positive feedback and restoring cellular homeostasis

To counter the upstream SCFA deficiency and the midstream organellar vicious cycle, exogenous supplementation of SCFAs (particularly butyrate) or the promotion of their endogenous production via dietary fiber or prebiotics represents a promising intervention strategy to interrupt the pathological positive feedback. SCFAs do not simply act on a single organelle; rather, they simultaneously modulate the two core nodes—mitochondria and the ER—by restoring the functional coupling between them, thereby progressively converting the pathological positive feedback into a negative feedback that stabilizes cellular homeostasis.

First, at the mitochondrial level, butyrate, acting as an HDAC inhibitor, activates the PGC-1α signaling pathway, promoting mitochondrial biogenesis, restoring ATP-generating capacity, stabilizing mitochondrial membrane potential, and reducing mPTP opening and ROS production (Wu et al., 2024; Sun et al., 2018). The restoration of ATP supply not only improves neuronal energy metabolism but also re-supplies the ER with the energy required for protein folding.

Second, at the ER level, SCFAs upregulate the expression of molecular chaperones such as GRP78, thereby enhancing protein-folding capacity, alleviating ER stress, modulating IP3R-mediated Ca^2+^ release, restoring ER Ca^2+^ store homeostasis, and reducing aberrant Ca^2+^ transfer to mitochondria (Lu et al., 2024a; Li et al., 2025).

Concurrently, SCFAs inhibit NF-κB inflammatory signaling via GPR41/GPR43 activation, thereby attenuating the dual insults of inflammatory mediators to both mitochondria and the ER (Wei et al., 2023; Ortiz-Samur et al., 2025; Wang Y. et al., 2025).

Collectively, these actions establish a novel functional cycle: Restoration of ATP → Improved ER protein folding → Restoration of Ca^2+^ homeostasis → Reduction of ROS → Further enhancement of mitochondrial function. This cycle, instead of amplifying injury, progressively reinforces cellular repair capacity, enabling a dynamic shift from pathological positive feedback to protective negative feedback. The improvement in mitochondrial function reduces ER calcium loading, while the alleviation of ER stress in turn diminishes mitochondrial Ca^2+^ overload. These mutually reinforcing processes form a virtuous cycle, collectively restoring neuronal homeostasis, ultimately reducing apoptosis and exerting neuroprotective effects.

## 6 Conclusion

In summary, the neuropathology of neonatal HIE is not driven by a single molecular event, but is dominated by a vicious cycle between mitochondrial dysfunction and ER stress. This positive feedback loop, physically coupled through MAMs, tightly links calcium overload, ROS burst, and energy depletion: ER stress leads to mitochondrial calcium overload and mPTP opening, while mitochondrial dysfunction in turn exacerbates ATP depletion and oxidative microenvironment disruption within the ER, shifting the UPR from an adaptive to a pro-apoptotic mode, ultimately culminating in irreversible neuronal loss via the CHOP-caspase-12-caspase-9 cascade and MOMP. Therefore, we propose that the “mitochondrial dysfunction–ER stress vicious cycle” represents the core mechanism of neuronal injury in HIE.

In this context, SCFAs, particularly butyrate, exhibit significant potential as a novel class of neuroprotective agents, owing to their unique advantages of multi-target action, natural origin, and favorable safety profile. Rather than acting as single-pathway drugs, SCFAs simultaneously target both mitochondria and the ER through three major mechanisms—HDAC inhibition, activation of GPR41/GPR43 receptors, and metabolic modulation. On one hand, butyrate epigenetically upregulates PGC-1α to promote mitochondrial biogenesis, stabilizes membrane potential, inhibits mPTP opening, and attenuates ROS production from the respiratory chain. On the other hand, by upregulating ER chaperones such as GRP78 and inhibiting overactivation of the PERK-eIF2α-ATF4-CHOP pathway, SCFAs effectively alleviate ER stress, shifting the UPR from a pro-apoptotic to an adaptive mode. More critically, SCFAs directly intervene in aberrant calcium signaling at MAMs, blocking the flow of signals from ER stress to mitochondrial damage. Simultaneously, improved mitochondrial function supplies ATP to the ER and clears ROS, thereby reversing the positive feedback into negative feedback. This “two-birds-with-one-stone” synergistic effect enables SCFAs to significantly reduce infarct volume, suppress neuroinflammation, protect the blood–brain barrier, and improve long-term cognitive function in animal models of HIE.

Nevertheless, translation from the bench to the neonatal intensive care unit (NICU) still faces multiple challenges. First, SCFAs (especially butyrate) have an extremely short half-life (minutes) *in vivo*, along with a strong odor and certain local irritancy; thus, sustained-release formulations or prodrugs suitable for neonates (e. g., tributyrin, sodium butyrate inclusion complexes) need to be developed. Second, the optimal route of administration and therapeutic time window remain undefined: although the core injury of HIE occurs during the 6–72 h post-reperfusion period, pharmacokinetic studies are required to determine whether SCFAs can effectively cross the blood–brain barrier under hypoxic conditions and what brain concentrations must be achieved. Third, although SCFAs are generally safe as endogenous metabolites, toxicological data regarding potential adverse effects due to accumulation in neonates with severe acidosis or hepatic/renal insufficiency are still lacking. Finally, clinical studies must address inter-individual variability: the initial gut microbiota composition, feeding patterns (breast milk vs. formula), and history of antibiotic exposure all affect endogenous SCFA levels; therefore, the dosage for exogenous supplementation may need to be adjusted based on “microbiota typing.”

Looking forward, translating the “gut–brain axis” theory into practical clinical intervention strategies should focus on the following directions. First, conduct large-animal HIE model studies to define the dosage, frequency, and efficacy-safety window of SCFAs or SCFA-producing probiotics (*Clostridium butyricum*, *Bifidobacterium infantis*). Second, design randomized controlled trials (RCTs) with primary endpoints including normalization rate on amplitude-integrated electroencephalography (aEEG), N-acetylaspartate/choline ratio on magnetic resonance spectroscopy (MRS), and neurodevelopmental scores at 18 months of age, to validate the synergistic effect of SCFAs combined with therapeutic hypothermia. Third, develop non-invasive monitoring strategies, such as real-time assessment of gut microbiota function via fecal SCFA profiling or breath hydrogen/methane concentrations, enabling individualized precision supplementation. Fourth, develop novel targeted delivery systems (e. g., nano-encapsulated butyrate or engineered probiotics) that enable SCFAs to cross the blood–brain barrier and release sustainably within the brain. Only through multidisciplinary collaboration that closely integrates basic mechanistic research with clinical translation can we ultimately break the vicious cycle in HIE and enable this natural gut-derived metabolite to truly benefit neonates at risk of lifelong brain injury.
